# Ultrasmall Glutathione-Protected Gold Nanoclusters as Next Generation Radiotherapy Sensitizers with High Tumor Uptake and High Renal Clearance

**DOI:** 10.1038/srep08669

**Published:** 2015-03-02

**Authors:** Xiao-Dong Zhang, Zhentao Luo, Jie Chen, Shasha Song, Xun Yuan, Xiu Shen, Hao Wang, Yuanming Sun, Kai Gao, Lianfeng Zhang, Saijun Fan, David Tai Leong, Meili Guo, Jianping Xie

**Affiliations:** 1 Tianjin Key Laboratory of Radiation Medicine and Molecular Nuclear Medicine, Institute of Radiation Medicine, Chinese Academy of Medical Sciences and Peking Union Medical College, No. 238, Baidi Road, Tianjin. 300192, China; 2 Department of Chemical and Biomolecular Engineering, National University of Singapore, 4 Engineering Drive 4, Singapore 117585, Singapore; 3 Key Laboratory of Human Disease Comparative Medicine, Ministry of Health, Institute of Laboratory Animal Science, Chinese Academy of Medical Sciences & Comparative Medical Center, Peking Union Medical College, Beijing. 100021, China; 4 Department of Physics, School of Science, Tianjin Chengjian University, Tianjin 300384, China

## Abstract

Radiotherapy is often the most straightforward first line cancer treatment for solid tumors. While it is highly effective against tumors, there is also collateral damage to healthy proximal tissues especially with high doses. The use of radiosensitizers is an effective way to boost the killing efficacy of radiotherapy against the tumor while drastically limiting the received dose and reducing the possible damage to normal tissues. Here, we report the design and application of a good radiosensitizer by using ultrasmall Au_29–43_(SG)_27–37_ nanoclusters (<2 nm) with a naturally-occurring peptide (e.g., glutathione or GSH) as the protecting shell. The GSH-coated Au_29–43_(SG)_27–37_ nanoclusters can escape the RES absorption, leading to a good tumor uptake (~8.1% ID/g at 24 h post injection). As a result, the as-designed Au nanoclusters led to a strong enhancement for radiotherapy, as well as a negligible damage to normal tissues. After the treatment, the ultrasmall Au_29–43_(SG)_27–37_ nanoclusters can be efficiently cleared by the kidney, thereby avoiding potential long-term side-effects caused by the accumulation of gold atoms in the body. Our data suggest that the ultrasmall peptide-protected Au nanoclusters are a promising radiosensitizer for cancer radiotherapy.

Cancer remains one of the world's most devastating diseases with more than 10 million new cases each year, and radiotherapy is a leading cancer treatment approach that addresses the needs of more than 50% cancer patients^1^. Though high-energy radiation can fatally damage tumor cells, it can also harm normal tissues. In fact, the mitotically active tumor cells are only slightly more susceptible to radiation damage than those in the essential normal tissues^2^. Hence, it is very important to strike the right balance between eradicating tumor and saving normal tissues by controlling the target and the dose of radiation administered to the patient. Many improvements have been made in radiotherapy to target tumors better, which could cause less damage to normal tissues. For example, megavolt (6–25 MV) X-rays are now used to avoid skin damage; tomotherapy and intensity-modulated radiation therapy (IMRT) are applied to better concentrate the radiation within the tumor volume; and optimal dose fractionation schedules are also developed to allow better cumulative damages to the tumor and adequate repairing of normal tissues^2^,^3^,^4^,^5^. Despite such advances, it is still challenging to use radiotherapy alone to eradicate tumor cells. A magic bullet to current challenges in radiotherapy is radiosensitizer, which can locally increase the efficacy of radiotherapy by enhancing the radiation damages to the cell.

In general, the radiosensitizing agents can be classified into two major categories according to their mechanisms of action: (type-1) chemotherapeutics that modulate the cell response to enhance the radiation damage, and (type-2) materials that interact directly with the radiation and generate additional damages to the cell^2^,^3^,^6^,^7^. The development of type-1 radiosensitizers started with Heidelberger's preclinical studies^8^ in 1958, and this radiosensitizing approach is often referred to as combined chemotherapy and radiotherapy or chemoradiation^3^,^9^. Most organic radiosensitizers are type-1, which enhance radiotherapy by modulating cell responses, such as reducing the radioresistance of tumor cells, preventing the formation of blood vessels (or disrupting the existing vessels, anti-angiogenic), inducing apoptosis, and suppressing mitosis^3^,^6^,^8^. Although many preclinical and clinical studies have affirmed the efficacy of type-1 radiosensitizers, a major drawback of these chemotherapeutics is their inherent cytotoxicity and side effects. For example, gemcitabine is known to cause myelosuppression, anemia, vomiting, and diarrhea^10^,^11^. Similarly, cisplatin is known to have myelotoxicity, neurotoxicity, and nephrotoxicity, and it can also cause hemolytic anemia, hearing loss, and vomiting^12^,^13^,^14^.

Type-2 radiosensitizers are mostly metal-based materials that can strongly absorb, scatter, and reemit radiation energy, resulting in a local radiation dose increase when they are accumulated in tumors^15^,^16^. Intense research on nanoscale metallic materials in the past two decades has provided many novel materials for biomedical applications^17^. Among these emerging radiosensitizers, gold nanoparticles (Au NPs) are particularly attractive because of their strong interaction with the radiation (Au has a high atomic number of 79), excellent chemical stability and inertness, and good biocompatibility (low toxicity)^18^,^19^,^20^,^21^. The enhancement of radiation dose received by the tumor tissue loaded with Au relative to the dose received by normal tissues without Au can be 200% or higher^22^,^23^. Such enhancement comes from the direct interaction between Au and radiation. When the incident radiation (gamma rays, X-rays) impinges on a Au NPs, the NPs becomes a new source of radiation and emits high energy through scattered photons (X-rays), photoelectrons, Compton electrons, Auger electrons, electron–positron pairs, and fluorescence photons, thereby causing radiochemical (free radicals and ionization) damages to the surrounding tumor tissue^22^,^24^,^25^. However, most of the Au NPs that have been demonstrated so far have large particle sizes (typically above 50 nm) and could be trapped by the reticulo-endothelial system (RES) absorption, which could result in low tumor uptake and unavoidable accumulation in liver and spleen^26^,^27^,^28^,^29^,^30^,^31^. Decreasing the particle size could benefit the escape of particles from the RES absorption. For example, one recent study showed that Au NPs with particle sizes below 20 nm could efficiently escape the RES absorption and showed good tumor uptake^32^. However, the sizes of these particles were still above the renal clearance barrier, that is ~5.5 nm, and could therefore induce the accumulation of NPs in RES, thus resulting in potential toxicity over the long term^33^,^34^,^35^,^36^. Besides the core size of NPs, the protecting ligands on the NPs surface can also affect the *in vivo* biodistribution. For example, the naked Au NPs of particle sizes of 1.9 and 4.8 nm, while small, have low colloidal stability due to the protein corona acquired in blood. These Au NPs eventually formed large aggregates of ~20–100 nm, which could not be rapidly metabolized and certainly unable to escape the RES^18^,^37^. Au NPs with different surface ligands can induce different NPs-protein corona in blood that could determine the RES absorption and cellular uptake efficiency^38^.

Taken together of the two key attributes (size and surface) for NPs-based radiosensitizers, we hypothesized that: 1) small naturally-occurring peptides, such as glutathione or GSH, could be a good surface ligand for Au NPs by helping them escape the RES absorption and improving their deposition in tumors; and 2) ultrasmall Au NPs with core sizes below 2 nm (hereafter referred to as nanoclusters, NCs) in combination with the GSH ligands can ensure a small hydrodynamic diameter (HD), which could provide good interface with the biological system, improve their *in vivo* pharmacokinetics, and enhance their deposition in tumors^39^. Here we demonstrate such concept by using sub-2-nm GSH-protected Au NCs with a well-defined molecular formula of Au_29–43_(SG)_27–37_^40^. We show in this study that the Au_29–43_(SG)_27–37_ NCs have attractive features of high tumor uptake, strong sensitizing enhancement for radiation, and low toxicity, and they could be a good candidate for next generation radiosensitizers for clinical use. This study has therefore enriched the family of Au NPs and NCs that could show good performance for cancer radiotherapy^33^,^37^.

## Results and discussion

**Figure 1:**
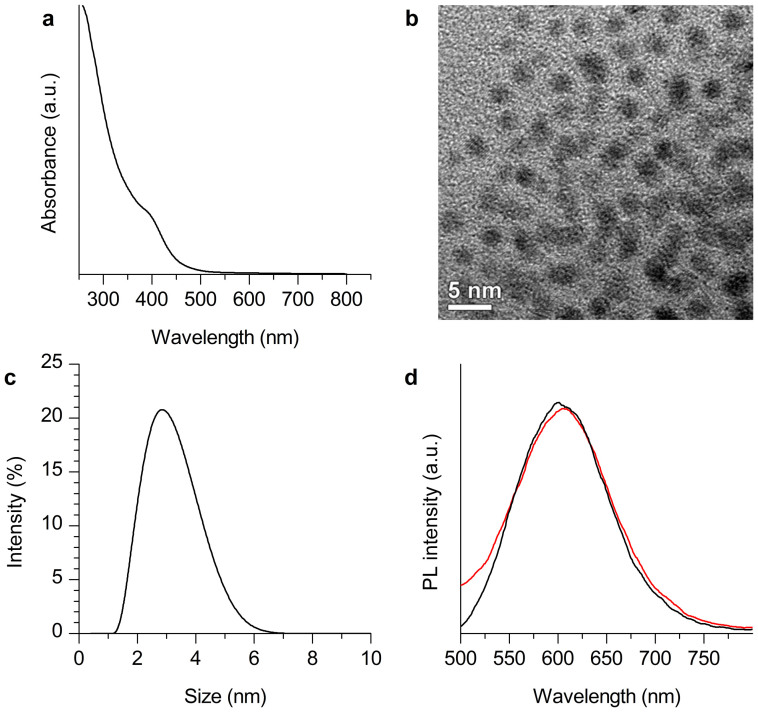
(a) UV-vis absorption spectrum, (b) TEM image, and (c) hydrodynamic diameter (measured by dynamic light scattering) of the as-prepared Au_29–43_(SG)_27–37_ NCs. (d) Photoluminescence spectra (λ_ex_ = 365 nm) of Au_29–43_(SG)_27–37 _NCs (black line) and the mixture of Au_29–43_(SG)_27–37_ NCs and blood plasma (at 24 h after mixing, red line).

The Au_29–43_(SG)_27–37_ NCs were prepared by a reported procedure^40^. The as-prepared Au NCs showed a shoulder peak at ~400 nm in the UV-vis absorption spectrum (Figure 1a), and surface plasmon resonance (SPR, typically at ~520 nm, a characteristic absorption of large Au NPs) was not observed. The molecular-like absorption of these Au NCs could be attributed to the discrete electronic states arising from the ultrasmall size of the NCs^40^,^41^,^42^,^43^,^44^,^45^. A representative transmission electron microscopy (TEM, Figure 1b) image confirmed that the Au NC cores were smaller than 2 nm. The hydrodynamic diameter (HD) of Au_29–43_(SG)_27–37_ NCs was determined to be ~2.8 nm by using dynamic light scattering (DLS, Figure 1c). In addition, Au_29–43_(SG)_27–37_ NCs showed strong orange luminescence with an emission peak at ~610 nm (Figure 1d, black line), which was also consistent with the previous report^40^.

We tested the blood stability of the as-prepared Au_29–43_(SG)_27–37_ NCs and the extent of plasma protein that binds to the NCs by missing Au_29–43_(SG)_27–37_ NCs (0.5 mL, 3 mM per Au atom) with blood plasma (0.5 mL). The photoluminescence of the mixture of Au_29–43_(SG)_27–37_ NCs and blood plasma (at 24 h after mixing) was not decreased significantly as compared with the aqueous solution of the NCs (Figure 1d), suggesting that Au_29–43_(SG)_27–37_ NCs were sufficiently stable in blood. The unbound Au_29–43_(SG)_27–37_ NCs were separated from the protein-bound Au NCs by filtering the mixture of Au_29–43_(SG)_27–37_ NCs and blood plasma (at 24 h after mixing) using ultrafiltration with a molecular weight cut-off, MWCO of 50 kDa. About 40% of Au_29–43_(SG)_27–37_ NCs were recovered from the filtrate as determined by their photoluminescence intensity (Figure S1), indicating that the binding ratio of plasma protein was ~60%.

**Figure 2:**
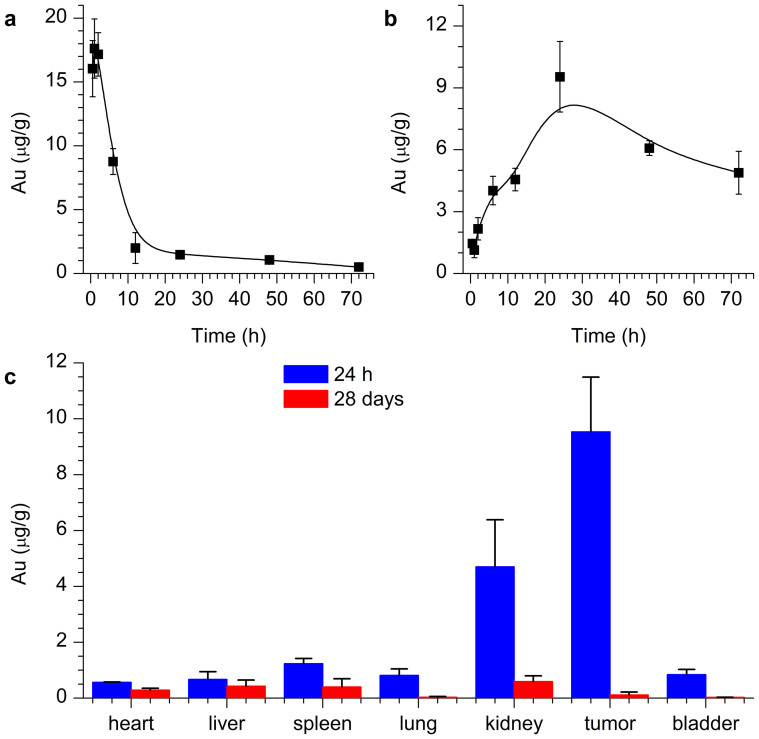
(a) *In vivo* blood concentration studies of Au_29–43_(SG)_27–37_ NCs. (b) Tumor uptake of Au_29–43_(SG)_27–37_ NCs after different time injection. (c) Biodistribution of Au_29–43_(SG)_27–37_ NCs after 24 h and 28 days p.i.

We further performed *in vivo* experiments to investigate the pharmacokinetics of the Au_29–43_(SG)_27–37_ NCs. The mice were intraperitoneally injected with the Au_29–43_(SG)_27–37_ NCs (~5.9 mg-Au/kg-body). As shown in Figure 2a, the distribution half-life (first phase t*_1/2α_*) of Au_29–43_(SG)_27–37_ NCs in blood was determined to be 6.5 h. As compared with the reported Au_10–12_(SG)_10–12_ and Au_25_(SG)_18_ NCs, the longer distribution half-life of the Au_29–43_(SG)_27–37_ NCs could be attributed to their larger hydrodynamic diameters^33^,^46^. The concentration of Au_29–43_(SG)_27–37_ NCs in blood was gradually stabilized after ~12 h (Figure 2a). The high concentration of Au_29–43_(SG)_27–37_ NCs in blood may lead to high tumor uptake of the NCs.

The tumor uptake of the Au_29–43_(SG)_27–37_ NCs was measured using inductively coupled plasma mass spectrometry (ICP-MS, Figure 2b). The tumor uptake of the Au NCs reached a maximum at 24 h post injection (p.i.), corresponding to 8.1% ID/g (9.5 μg/g). The tumor uptake gradually decreased from 24 to 48 h p.i. The observed tumor uptake was higher than that of the previously reported PEG-coated Au nanorods (~7.1% ID/g)^27^, Au NPs (~3% ID/g)^29^,^37^, small Au NCs (~2.3–3.2% ID/g)^47^. We recently reported two kind of clusters, Au_25_(SG)_18_ and Au_10–12_(SG)_10–12_, and their tumor uptake were determined to be 13% and 50% ID/g, respectively^33^,^46^. In general, smaller particles may feature with higher tumor uptake. Compared with Au_25_(SG)_18_ and Au_10–12_(SG)_10–12_, the tumor uptake of Au_29–43_(SG)_27–37_ is relatively lower. However, one salient point of Au_29–43_(SG)_27–37_ is its strong orange emission at 610 nm with a high quantum yield of 15%; such strong emission could be advantageous for some biomedical applications. The ratios of the concentration of Au in tumor relative to that in other tissues and organs are important parameters to evaluate the specificity of the NCs. The tumor/kidney, tumor/blood, and tumor/liver ratios were determined to be 2.1/1.0, 4.5/1.0, and 14.2/1.0, respectively.

Detailed biodistribution and clearance of Au_29–43_(SG)_27–37_ NCs were further investigated. Figure 2c shows the biodistributions of Au_29–43_(SG)_27–37_ NCs at 24 h and 28 days p.i. Tumor and kidney possessed predominant distributions relative to spleen, liver, heart, and lung at 24 h p.i., which supports that Au_29–43_(SG)_27–37_ NCs could escape RES absorption and achieve efficient targeting. The majority of Au were cleared at 28 days p.i. because only 0.2% ID/g Au in liver, ~0.4% ID/g Au in kidney, and <0.1% ID/g in tumor were found, suggesting a high efficacy of renal clearance of Au NCs^48^,^49^. In contrast, many other inorganic nanomaterials, such as Au NPs, carbon nanotubes, and graphene, are difficult to be cleared^28^,^37^,^50^,^51^. It is worth mentioning that the Au_29–43_(SG)_27–37_ NCs with GSH ligands on the NC surface featured with a different biodistribution from that of the Cy5-labeled Au_25_(SG)_18_^46^. The possible reason could be the Cy5 labeling, which might modify the surface chemistry of Au_25_(SG)_18_^52^. However, in the pristine Au_29–43_(SG)_27–37_ NCs, the GSH ligand on the NC surface may help mitigate the serum protein adsorption^53^.

**Figure 3:**
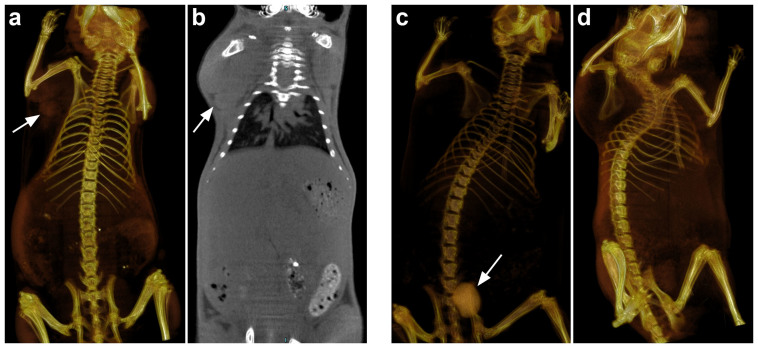
Small animal X-ray computed tomography (a) three-dimensional and (b) two-dimensional imaging of Au_29–43_(SG)_27–37_ NCs at 6 h p.i. using U14 tumor bearing mice. Renal clearance of Au_29–43_(SG)_27–37_ NCs at the time point of (c) 1 h and (d) 24 h p.i. using nude mice without tumor.

We also confirmed the tumor uptake and efficient renal clearance of Au_29–43_(SG)_27–37_ NCs by the X-ray computed tomography *in vivo* imaging (Figure 3). X-ray CT imaging is a non-invasive and reliable method for tumor imaging. The CT signal depends on the concentration of Au in tissues. A CT value of 1212 HU corresponding to 60 mM of Au (Figure S2), which is a good value for *in* *vivo* imaging. In this study, the as-prepared Au_29–43_(SG)_27–37_ NCs (60 mM Au, 0.2 mL) were injected into mice *via* tail vein, and two-and three-dimensional X-ray CT images were recorded. We measured the tumor uptake of Au_29–43_(SG)_27–37_ NCs using U14 tumor bearing mice. As shown in Figure 3a and 3b, the corresponding CT value was determined to be 365 HU, which was much higher than that of the muscle tissue (214 HU). A significant tumor uptake was observed in the tumor site (indicated by the arrows, Figure 3a) at 6 h p.i. In addition, a clear boundary between tumor and normal tissue was observed. Figure 3c and 3d showed the renal clearance of Au_29–43_(SG)_27–37_ NCs at the time points of 1 and 24 h p.i., measured using nude mice without tumor. The bladder (indicated by the arrow, Figure 3c) showed high contrast at 1 h p.i. (1300 HU), and this value (383 HU) was obviously decreased at 24 h p.i., indicating the efficient clearance of Au_29–43_(SG)_27–37_ NCs by kidney^49^.

**Figure 4:**
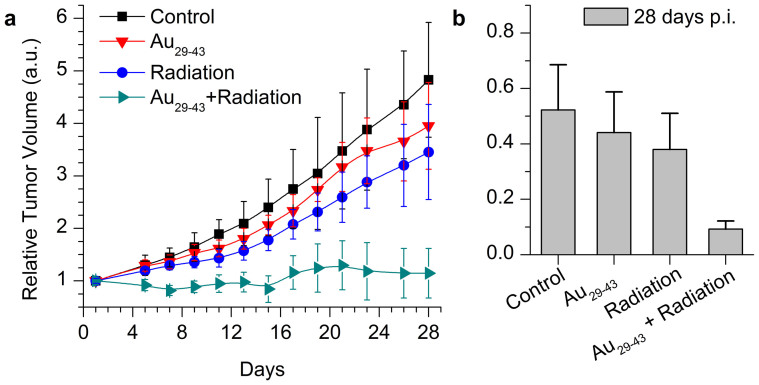
Time-course studies of tumor (a) volume and (b) weight of mice treated with Au_29–43_(SG)_27–37_ NCs at the concentration of 5.9 mg-Au/kg-body.

We also examined the cancer radiation treatment of Au_29–43_(SG)_27–37_ NCs by using U14 tumor bearing nude mice as the animal model. The mice were intraperitoneally injected with Au_29–43_(SG)_27–37_ NCs of a concentration of 5.9 mg-Au/kg-body. As a maximum tumor uptake of Au_29–43_(SG)_27–37_ NCs was reached at 24 h p.i. (Figure 2b), the mice were irradiated under ^137^Cs gamma radiation of 3600 Ci at a 5 Gy dose at 24 h p.i. At 28 days p.i., the tumor volumes and weights in the sacrificed mice were measured (Figure 4a). Compared with the control group, a remarkable decrease (~76%) of tumor volume was observed in mice treated with Au_29–43_(SG)_27–37_ NCs plus radiation (p < 0.05). In addition, compared with the mice treated by radiation only, the tumor volume decreased to ~66% in mice treated with Au_29–43_(SG)_27–37_ NCs plus radiation (p < 0.05). Figure 4b showed that the tumor weight decreased in mice treated with Au_29–43_(SG)_27–37_ NCs plus radiation. Similarly, a significant tumor weight decrease was seen in mice treated with Au_29–43_(SG)_27–37_ NCs plus radiation relative to that in mice treated with radiation only, suggesting that the Au_29–43_(SG)_27–37_ NCs can enhance the radiation therapy.

**Figure 5:**
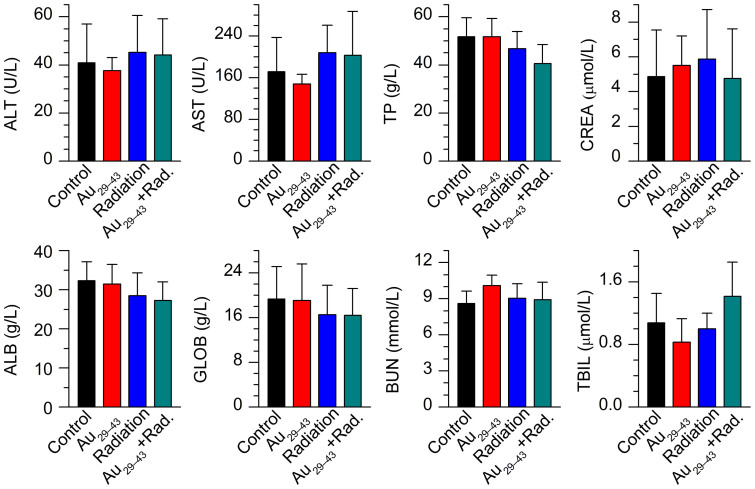
Blood biochemistry analysis of mice treated with Au_29–43_(SG)_27–37_ NCs at 28 days p.i. The results show mean and standard deviation of alanine aminotransferase (ALT), aspartate aminotransferase (AST), total protein (TP), albumin (ALB), blood urea nitrogen (BUN), creatinine (CREA), globulin (GOLB), and total bilirubin (TB).

**Figure 6:**
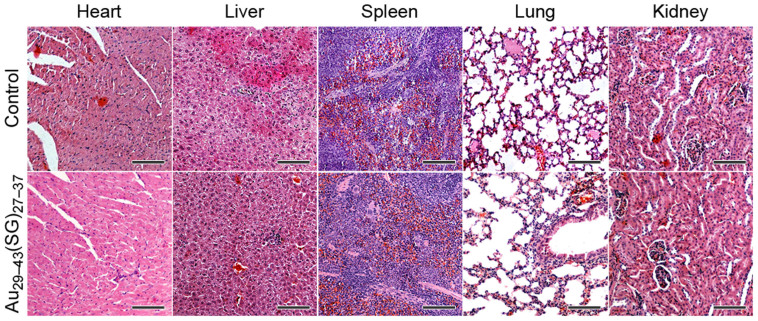
Pathological data from the heart, liver, spleen, lung, and kidney of mice treated with Au_29–43_(SG)_27–37_ NCs at the concentration of 5.9 mg-Au/kg-body. Scale bars, 100 μm.

We finally checked the toxicological responses by examining blood biochemistry (Figure 5) and pathology (Figure 6) of the mice. No significant weight loss, drastic organ or blood chemistry changes were found, suggesting that the renal clearable Au_29–43_(SG)_27–37_ NCs did not induce a significant liver and kidney toxicity. In contrast, the naked Au NPs, PEG-coated Au NPs, and BSA-protected Au NCs with the hydrodynamic diameter of ~6–100 nm have been found with acute liver toxicity, such as the increase of alanine aminotransferase (ALT) and aspartate aminotransferase (AST)^37^,^50^,^54^,^55^,^56^. Traditional radiosensitizers, such as cisplatin, also showed high kidney toxicity due to slow clearance^57^. Thus, the Au_29–43_(SG)_27–37_ NCs developed in this study could emerge as an attractive radiosensitizing agent with its low toxicity and high tumor uptake.

## 

In summary, the Au_29–43_(SG)_27–37_ NCs covered by GSH can escape the RES absorption and showed high tumor accumulation *via* the improved EPR effect. The hydrodynamically ultrasmall Au_29–43_(SG)_27–37_ NCs showed very efficient renal clearance, and no obvious toxicity was observed in the body. The as-designed Au NCs can also significantly enhance the efficacy of the cancer radiotherapy. These advantageous features allow the Au_29–43_(SG)_27–37_ NCs to be attractive radiosensitizer materials for further testing.

## Methods

### Synthesis and characterizations of Au_29–43_(SG)_27–37_ NCs

The synthesis and purification of Au_29–43_(SG)_27–37_ NCs followed the published procedures^40^,^58^. Briefly, freshly prepared aqueous solutions of HAuCl_4_ (20 mM, 0.50 mL) and GSH (100 mM, 0.15 mL) were mixed with 4.35 mL of ultrapure water at 25°C. The reaction mixture was heated to 70°C under gentle stirring (500 rpm) for 24 h. An aqueous solution of intensely orange-emitting Au_29–43_(SG)_27–37_ NCs was formed. The orange-emitting Au_29–43_(SG)_27–37_ NC solution could be stored at 4°C for 6 months with negligible changes in their optical properties. The as-prepared Au_29–43_(SG)_27–37_ NCs were purified through ultrafiltration (3 kDa membrane).

### *In vivo* biodistribution

The studies were approved by the Institute of Radiation Medicine, Chinese Academy of Medical Sciences and Animal Care Research Advisory Committee of Institute of Radiation Medicine, Chinese Academy of Medical Sciences, while experiments conducted following the guidelines of the Animal Research Ethics Board of Institute of Radiation Medicine, Chinese Academy of Medical Sciences. Forty-eight mice were purchased, maintained, and handled using protocols approved by the Institute of Radiation Medicine, Chinese Academy of Medical Sciences (CAMS). The U14 tumor models were generated by subcutaneous injection of 2 × 10^6^ cells suspended in 50 μL of PBS into the right shoulder of male nude mice. The mice treated with Au_29–43_(SG)_27–37_ NCs were sacrificed at 0.5, 1, 2, 6, 12, 24, 48, and 72 h post injection (p.i.). The main organs, such as tumor, liver, kidney, spleen, heart, lung, brain were collected. The organs of Au_29–43_(SG)_27–37_ NCs treated mice were digested using a microwave system CEM Mars 5 (CEM, Kamp Lintfort, Germany) to determine their Au content, which was determined by an inductively coupled plasma mass spectrometer (Agilent 7500 CE, Agilent Technologies, Waldbronn, Germany).

### *In vivo* imaging

Eighteen mice were purchased, maintained, and handled using protocols approved by the Institute of Radiation Medicine, Chinese Academy of Medical Sciences (CAMS). The U14 tumor models were generated by subcutaneous injection of 2 × 10^6^ cells suspended in 50 μL of PBS into the right shoulder of male nude mice. Before the experiments, the mice were anesthetized by chloral hydrate. For CT imaging, 200 μL of GSH-protected Au_29–43_(SG)_27–37_ NCs (60 mM, 0.2 mL) were injected through the intraperitoneal routes into mice. Each mouse was imaged on a small-animal scanner (microPET/CT, Inveon, Siemens). The mice were exposed to a 10-min CT scan and the images were reconstructed using the filtered back-projection algorithm with CT-based photon-attenuation correction. CT data were analyzed for regions of interest, including tumor, bladder, and spleen.

### *In vivo* radiation therapy

All animals were purchased, maintained, and handled using protocols approved by the Institute of Radiation Medicine, CAMS. The U14 tumor models were generated by subcutaneous injection of 2 × 10^6^ cells suspended in 50 μL of PBS into the right shoulder of BALB/c mice. The male mice were intraperitoneally treated with the Au_29–43_(SG)_27–37_ NCs when the tumor volume reached 100–120 mm^3^ (7 days after tumor inoculation). For each treatment, Au_29–43_(SG)_27–37_ NCs (0.59 mg-Au/mL) were intraperitoneally injected at a dosage of 5.9 mg/kg in the mice. As the control, 200 μL of saline was intraperitoneally injected into each mouse in the control group. Subsequently, the mice were irradiated by 5 Gy gamma-rays from ^137^Cs (photon energy 662 keV) with an activity of 3600 Ci at 24 h p.i. for Au_29–43_(SG)_27–37_ NCs injections. Thirty two male mice were assigned to the following four groups (eight mice per group): control, Au_29–43_(SG)_27–37_, radiation alone, and Au_29–43_(SG)_27–37_ + radiation. The tumor size was measured every two or three days, and calculated using the equation: tumor volume = (tumor length) × (tumor width)^2^/2.

### *In vivo* toxicity

The treated mice were weighed and assessed for behavioral changes. All mice were sacrificed at 28 days p.i., and their blood and organs were collected for hematology, biochemistry and toxicological investigation. The blood was drawn for hematology analysis (potassium EDTA collection tube) and serum biochemistry analysis (lithium heparin collection tube) using a standard saphenous vein blood collection technique. During necropsy, liver, kidney, spleen, heart, lung, brain, genitals, tumor, and thyroid were collected and weighed. Major organs from these mice were then fixed in 4% neutral buffered formalin, processed into paraffin, and stained with hematoxylin and eosin (H&E). Pathology was examined using a digital light microscope.

## Author Contributions

X.Z., Z.L., J.X. and M.G. conceived the project and designed the experiments. J.C., Z.L., X.S., S.S., X.Y. and X.Z. performed the experiments. Z.L., H.W. and X.Y. synthesized the materials and J.C., X.S., L.Z., K.G., Y.S. and S.S. performed the *in vivo* experiment. X.Z., Z.L., S.F., D.T.L. and J.X. analyzed the data and co-wrote the paper. All authors discussed the results and commented on the manuscript.

## Supplementary Material

Supplementary Information SI
