# Supplementary material for: Ultrasmall Glutathione-Protected Gold Nanoclusters as Next Generation Radiotherapy Sensitizers with High Tumor Uptake and High Renal Clearance
Source: Sci Rep. 2015 Mar 2;5:8669. doi: 10.1038/srep08669 (PMC4345316; doi:10.1038/srep08669)
Supplement: Supplementary Information — SI [file srep08669-s1.pdf]

SUPPORTING INFORMATION

Ultrasmall Glutathione-Protected Gold Nanoclusters as Next Generation Radiotherapy Sensitizers with High Tumor Uptake and High Renal Clearance

Xiao-Dong Zhang,[a] Zhentao Luo,[b] Jie Chen,[a] Shasha Song,[a] Xun Yuan,[b] Xiu Shen,[a] Hao Wang,[a] Yuanming Sun,[a] Kai Gao,[c] Lianfeng Zhang,[c] SaijunFan,[a] David Tai Leong,[b] MeiliGuo,[d*] and Jianping Xie[b*]

[a] Tianjin Key Laboratory of Radiation Medicine and Molecular Nuclear Medicine, Institute of Radiation Medicine, Chinese Academy of Medical Sciences and Peking Union Medical College, No. 238, Baidi Road, Tianjin, 300192, China

[b] Department of Chemical and Biomolecular Engineering,National University of Singapore, 4 Engineering Drive 4, Singapore 117585 (Singapore)

[c] Key Laboratory of Human Disease Comparative Medicine, Ministry of Health, Institute of Laboratory Animal Science, Chinese Academy of Medical Sciences & Comparative Medical Center, Peking Union Medical College, Beijing, 100021, China

[d] Department of Physics, School of Science, Tianjin Chengjian University, Tianjin 300384, China

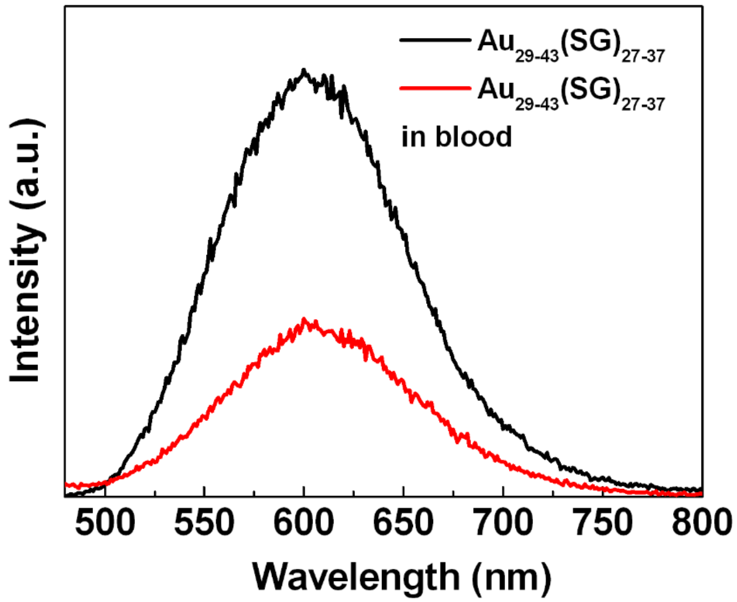

Figure S1. Photoluminescence spectra (λ_{ex} = 365 nm) of $Au_{29–43}(SG)_{27–37}$ NCs (black line) and the filtrate (red line) of the mixture of $Au_{29–43}(SG)_{27–37}$ NCs and blood plasma (at 24 h after mixing) by using ultrafiltration with a molecular weight cut-offof 50 kDa.

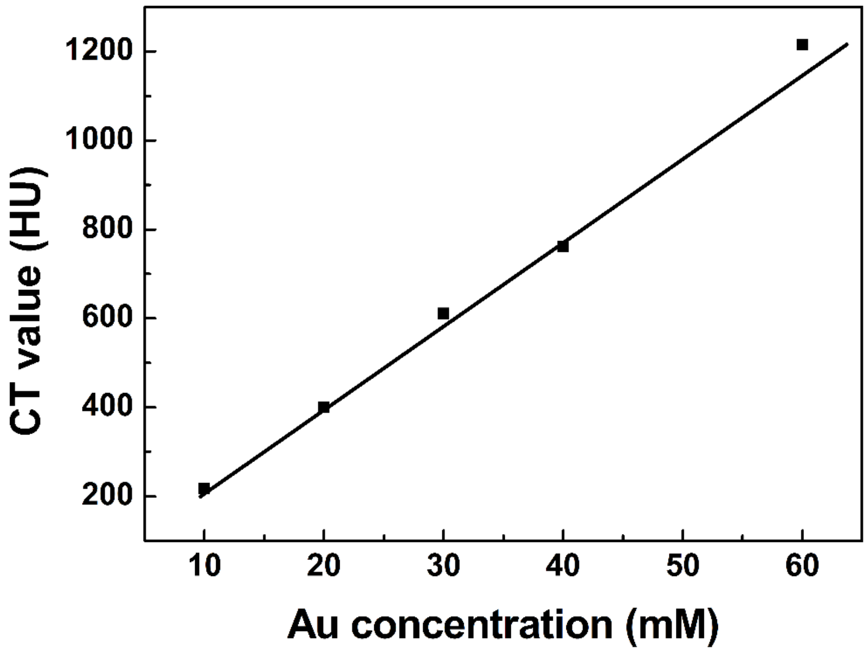

Figure S2. Correlation of CT value of $Au_{29\text{-}43}(SG)_{27\text{-}37}$NCs with Au concentration.
